# The feasibility and acceptability of research magnetic resonance imaging in adolescents with moderate–severe neuropathic pain

**DOI:** 10.1097/PR9.0000000000000807

**Published:** 2020-01-21

**Authors:** Madeleine Verriotis, Massieh Moayedi, Clarissa Sorger, Judy Peters, Kiran Seunarine, Christopher A. Clark, Suellen M. Walker

**Affiliations:** a Pain Research, Developmental Neurosciences, UCL Great Ormond Street Institute of Child Health, London, United Kingdom; b Department of Anaesthesia and Pain Management, Great Ormond Street Hospital NHS Foundation Trust, London, United Kingdom; c Centre for Multimodal Sensorimotor and Pain Research, University of Toronto, Toronto, ON, Canada; d Faculty of Dentistry, University of Toronto, Toronto, ON, Canada; e University of Toronto Centre for the Study of Pain, Toronto, ON, Canada; f Developmental Imaging and Biophysics Section, Developmental Neurosciences, UCL Great Ormond Street Institute of Child Health, London, United Kingdom

**Keywords:** Pain, Neuropathic pain, Children, Adolescents, Magnetic resonance imaging

## Abstract

**Introduction:**

Multimodal characterisation with questionnaires, Quantitative Sensory Testing (QST), and neuroimaging will improve understanding of neuropathic pain (NeuP) in adolescents. Magnetic resonance imaging (MRI) data in adolescents with NeuP are limited, and the perceived practical or ethical burden of scanning may represent a barrier to research.

**Objective:**

To determine the feasibility of MRI scanning in adolescents with moderate–severe NeuP, with respect to consent rate, postscan acceptability, and data quality.

**Methods:**

This prospective cohort study evaluating questionnaires and QST recruited adolescents aged 10 to 18 years with clinically diagnosed NeuP from a tertiary clinic. Eligible adolescents aged 11 years and older could additionally agree/decline an MRI scan. After the scan, families rated discomfort, perceived risk, and acceptability of current and future MRI scans (0–10 numerical rating scales). Head motion during scanning was compared with healthy controls to assess data quality.

**Results:**

Thirty-four families agreed to MRI (72% recruitment), and 21 adolescents with moderate–severe pain (average last week 6.7 ± 1.7; mean ± SD) and with neuropathic QST profiles were scanned. Three adolescents reported positional or noise-related discomfort during scanning. Perceived risk was low, and acceptability of the current scan was high for parents (range [median]: 7 to 10/10 [10]) and adolescents (8–10/10 [10]). Willingness to undergo a future research scan was high for parents (7–10/10 [10]) and adolescents (5–10/10 [10]) and did not differ from future scans for clinical purposes. Mean head motion during resting state functional MRI did not differ from control adolescents.

**Conclusion:**

Research MRI is feasible and acceptable for many adolescents with moderate–severe NeuP.

## Abstract 2

Supplemental Digital Content is Available in the Text.

## 1. Introduction

In adults with complex pain, detailed phenotyping with patient-reported outcome measures (PROMs), Quantitative Sensory Testing (QST), and neuroimaging improve patient stratification for clinical trials and treatment and provide mechanistic insight.^6–8,36^ In adolescents, neuropathic pain (NeuP) is associated with significant pain and pain-related disability,^18^ but causes can differ from adults, and evidence from paediatric trials is limited.^10^ Ongoing innovation in paediatric pain research with translation into clinical practice is needed.^3^

Although PROMs and QST have been used for a range of chronic pain conditions in highly symptomatic adolescents, relatively few studies have used MRI in adolescents with NeuP.^2,12,13,19,20,30,31^ Lack of evidence regarding feasibility and practical or ethical burden of MRI in such cohorts^28^ may represent barriers to research study planning, ethical approval, and/or recruitment.^33^ Within a larger clinical cohort of adolescents with moderate–severe NeuP, a pilot study assessed MRI consent rate, postscan acceptability, and data quality.

## 2. Methods

### 2.1. Participants

Adolescents aged 10 to 18 years with clinically diagnosed NeuP were recruited from the Great Ormond Street Hospital Chronic Pain Management Service. The MRI pilot forms part of an ongoing cohort study evaluating PROMs and QST (clinicaltrials.gov NCT03312881). Written informed parental consent and adolescent assent/consent were obtained, and families were given the option to additionally consent to an MRI scan, which required 1 additional hospital visit within 3 months of QST testing and recruitment (see Text, Supplemental Digital Content 3, which contains further recruitment details, available at http://links.lww.com/PR9/A60). Age-matched healthy participant data with the same MRI protocol and scanner were available for comparison.

### 2.2. Measures

#### 2.2.1. Pain intensity

At recruitment, adolescents completed visual analogue scales (VASs; 0–10 cm) for pain intensity (now, average and worst pain in the last week) and activity interference due to pain.^40^ Twelve adolescents also reported pain intensity immediately before MRI.

#### 2.2.2. Patient-reported outcome measures

Validated questionnaires completed during clinic appointments included: Pediatric Index of Emotional Distress^21^; Paediatric Quality of Life Inventory^38^; and Pain Catastrophizing Scale—Children.^35^

After the scan, adolescents and parent(s) rated discomfort, perceived risk, and acceptability of current and future MRI scans (0–10 numerical rating scale [NRS]) (see Figures, Supplemental Digital Content 1–2, which contain postscan questionnaires completed by participants, available at http://links.lww.com/PR9/A60).

#### 2.2.3. MRI acquisition and analysis

Multimodal neuroimaging was performed using a 3T Siemens Prisma MRI scanner with a 64-channel coil at Great Ormond Street Hospital. Neuroimaging included T1- and diffusion-weighted images and resting-state functional MRI (rsfMRI; see Text, Supplemental Digital Content 3, which provides MRI acquisition parameters and analysis methods, available at http://links.lww.com/PR9/A60). For the rsfMRI scan, participants were asked to keep their eyes closed and let their minds wander. Given our paediatric cohort, the protocol was restricted to 30 minutes.

As head motion can impair quality of fMRI,^15^ framewise displacement (FD)^24^ was measured as the movement of any given frame relative to the previous frame. Scans underwent standard preprocessing (see Text, Supplemental Digital Content 3, which provides MRI acquisition parameters and analysis methods, available at http://links.lww.com/PR9/A60) in the CONN toolbox (v18a),^42^ run on MATLAB (R2018a v9.4; Mathworks, Nantick, MA).

Framewise displacement values were compared between adolescents with NeuP and controls. As thresholds of 0.2 and 0.5 mm have been suggested to indicate high levels of motion in adults,^24,25^ we calculated the proportion of frames per participant above these thresholds.

### 2.3. Data analyses

Statistical analysis was performed with SPSS (v24; IBM, Portsmouth, United Kingdom). When assumptions of normality were not met, nonparametric tests were used. All tests were 2-tailed and assessed at α = 0.05.

## 3. Results

### 3.1. Participants

**Figure 1.**
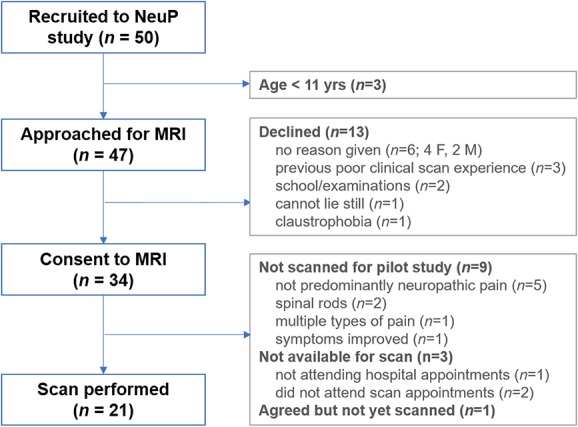
Recruitment flow chart for pilot MRI study in adolescents with a clinical diagnosis of neuropathic pain (NeuP). Ten- to 18-year-old patients (n = 50) were recruited to a study characterizing NeuP in adolescents using Quantitative Sensory Testing and patient-reported outcome measures. At the time of recruitment to the NeuP study, adolescents aged 11 years and older were additionally given the option to consent to a research MRI scan. After consent, participants were screened for suitability for the MRI portion of the study (see Text, Supplemental Digital Content 3, which contains further information relating to recruitment procedures, available at http://links.lww.com/PR9/A60), and an MRI appointment was arranged for eligible participants. F, female; M, male.

Fifty adolescents with NeuP (n = 42) or predominantly NeuP (n = 8) were recruited to the NeuP study between October 2017 and April 2019 (Fig. 1).

### 3.2. Pain ratings and patient-reported outcome measures

At recruitment, average pain intensity in the last week was moderate–severe in both males (mean ± SD: 6.2 ± 1.5; n = 19) and females (6.5 ± 2.2; n = 31). Participants indicated high pain catastrophizing and emotional distress and impaired quality of life (Table 1).

**Table 1:**
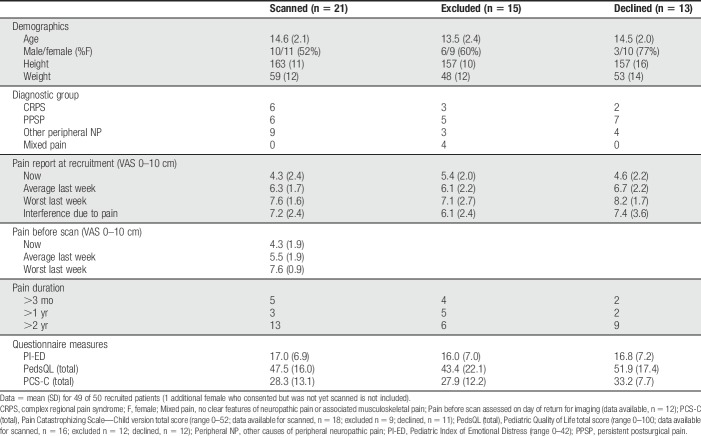
Comparative demographic, pain report, and questionnaire data for subgroups of patients recruited to the neuropathic pain study, who were scanned, consented to MRI but were excluded from the pilot study, or who declined an MRI scan.

### 3.3. MRI recruitment

Thirty-four of 47 (72%) adolescents aged 11 years and older and their families agreed to MRI. To reduce heterogeneity, we further excluded patients without neuropathic QST profiles of sensory gain/loss^1,29^ and those with multiple types of pain that could limit attribution of MRI changes to current NeuP (Fig. 1; see also Text, Supplemental Digital Content 3, which contains further exclusion details, available at http://links.lww.com/PR9/A60). Demographics, pain, and questionnaire measures in scanned patients did not differ from those who were excluded or declined MRI (Table 1). A higher but statistically insignificant proportion of females than males (10/30 vs 3/19) declined MRI.

### 3.4. Postscan acceptability and discomfort

**Figure 2.**
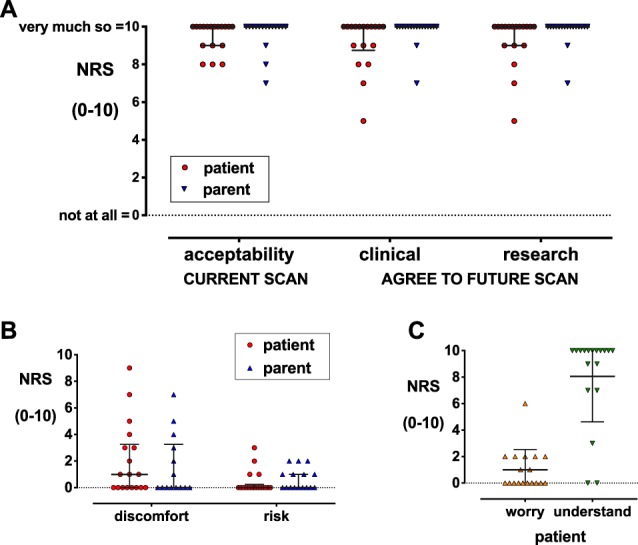
Experience and acceptability ratings after brain neuroimaging completed by adolescents and parents. Agreement was based on numerical rating scales (NRSs) from zero “not at all” to 10 “very much so.” (A) Adolescent and parent ratings for the current MRI and willingness to agree to a future scan for clinical or research purposes. (B) Adolescent and parent ratings for child's discomfort during the scan and perceived risk of MRI (C) Adolescent rating of level of worry and ability to understand instructions during the scan. Data points = individual values; bars = median (IQR).

Eighteen adolescents (10 female and 8 male) and 17 parents (1 declined as limited English) completed post-MRI questionnaires. Three parents felt unable to report child discomfort as they were not in the scanner room. Ratings for current research scan acceptability were high for both adolescents (range [median]: 8-10 [10]; 67% rated 10/10; “Overall, do you think it is ok for a brain scan to be performed to help understand "nerve" pain in children?”; see Figure, Supplemental Digital Content 1, which contains the postscan questionnaire completed by adolescents, available at http://links.lww.com/PR9/A60) and parents (7–10 [10]; 81% 10/10) (Fig. 2A). Acceptability of a future research scan was high for parents (7–10 [10]; 88% 10/10) but lower for adolescents (5–10 [10]; 67% 10/10) and did not differ from acceptability for future clinical scans (Fig. 2A).

Three adolescents declined MRI due to noise or discomfort during previous clinically required scans. Of 21 adolescents scanned for this study, 18 were asked to complete postscan questionnaires. Eight reported no discomfort, 6 mild discomfort (1–3/10), and 2 moderate (5–7/10) positional discomfort in the head or neck during MRI. One adolescent with 9/10 discomfort due to noise also reported the highest worry (6/10) and lowest acceptability of future research scans (5/10) (Fig. 2B, C). Within this small cohort, there was no correlation between pain intensity immediately before scanning and discomfort (Spearman's ρ = 0.13, *P* = 0.7; n = 12) or between previously completed PI-ED scores and worry during MRI (ρ = 0.33, *P* = 0.18; n = 18). Fifteen adolescents felt scan instructions were easy to understand (7–10/10, 61% 10/10). Two adolescents reporting difficulty understanding instructions (0/10) also had lower ratings for future scan acceptability (5–7/10) (Fig. 2C).

### 3.5. MRI data quality

**Figure 3.**
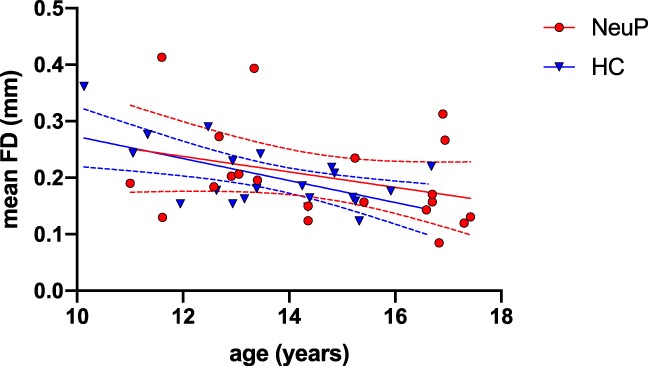
Mean head motion plotted against age for adolescents with neuropathic pain (NeuP) and age-matched healthy controls (HC). There was a negative relationship between age and mean framewise displacement (FD) across groups (Spearman's ρ = −0.39, *P* = 0.01, n = 42) and a trend in both subgroups (NeuP: ρ = −0.35, *P* = 0.12, n = 21; HC: ρ = −0.43, *P* = 0.05, n = 21). Data points = individual values; continuous lines = regression between age and mean FD per group; dotted lines = 95% confidence intervals.

Head motion during rsfMRI in NeuP patients did not differ from age-matched healthy controls (Table 2). Mean FD and the percentage of frames per adolescent with FD greater than either 0.2 or 0.5 mm were similar (Table 2), and there was a similar negative relationship between age and mean FD across both groups (Fig. 3).

**Table 2:**
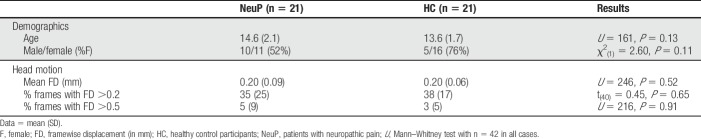
Comparative demographic and head motion data for patients and control participants who had a resting state fMRI scan.

## 4. Discussion

Many adolescents with moderate–severe NeuP and families agreed to research MRI and reported high acceptability of the current and future scans. Logistical issues and MRI contraindications accounted for some refusals. Previous poor scan experience influenced recruitment, and adolescents reporting discomfort or difficulty understanding instructions also had lower ratings for future scan acceptability. Providing families with information about other children's scan experience may facilitate decisions regarding recruitment.^32^

Neuroimaging pain research is well-established in adults,^7,36^ but additional pediatric data are required. Nociceptive processing is developmentally regulated and sensitive to early life experience,^4,39,40^ and correction for significant age and sex-dependent changes in brain structure throughout adolescence^5^ is needed when assessing disease effects.^34^ MRI has identified altered brain structure and function in adolescents with complex regional pain syndrome,^2,12,13,19,20,30,31^ but evaluations of acceptability and feasibility, and in other NeuP cohorts, are limited. Despite experiencing persistent moderate–severe NeuP with high levels of emotional distress and pain catastrophizing, recruitment and parental and adolescent acceptability of research MRI was high.

There is no gold standard for measuring research procedural discomfort in children.^33^ Although not formally validated, our numerical scales and questions regarding discomfort, anxiety, or concerns about the procedure, and willingness to undergo future scans, parallel those used for MRI acceptability in adults^14,22^ and child discomfort during research procedures.^32,33^ As suggested, both adolescent and parental self-report was obtained immediately after the procedure to minimize recall bias.^33^ Although overall satisfaction with clinically required scans despite discomfort may be heightened by perceived diagnostic value,^22^ adolescents and parents did not differentiate between acceptability of future scans for clinical or research purposes.

Data regarding the type and degree of discomfort during research procedures in adolescents can aid ethics committee evaluations of potential burden.^33,41^ Unsedated healthy participants aged 8 to 18 years undergoing research MRI for 30 to 60 minutes reported low overall discomfort (1.6 ± 0.45, mean ± SD; 1–5 Likert scale).^32^ Our data mirror these findings: Despite chronic NeuP, most adolescents tolerated MRI with minimal discomfort.

Feasibility of research MRI in adolescents also depends on obtaining high-quality data within a tolerable duration. Pediatric and clinical populations may be more susceptible to head motion and movement artefact,^23^ and removing affected data frames can result in loss of 50% or more of data^9^ and adversely affect interpretation.^24,25,27,37^ Others suggest that head motion is heritable and stable over time^11,17^ and also reflects individual variability in functional organization.^26,43^ Real-time visual feedback can reduce head movement in younger patients,^16^ and motion analytics can facilitate scanning until the desired amount of low-movement data has been collected.^9^ With our 30-minute scan protocol, head motion tended to be higher at younger ages as previously reported,^11,27^ but did not differ between clinical and healthy adolescents, and data were high-quality.

Behavioral strategies can improve acceptability and tolerability of MRI for unsedated adolescents,^16^ and adequate preparation can reduce anticipated pain or worry.^33^ Despite high pain and anxiety scores, worry during MRI was low, with experienced pediatric radiographers providing age-appropriate instructions throughout scanning and maximizing comfort during positioning. In accordance with adolescent preferences during research procedures,^16,32^ participants viewed a movie of his/her choice, apart from during rsfMRI. Advances in neuroimaging that reduce scan time will further improve tolerability for adolescents.

## 5. Limitations

The number of adolescents scanned for this pilot study is small (n = 21), and the MRI acceptability questionnaire was introduced after the first 3 participants. Acceptability ratings do not account for potential lower scores in 3 participants who declined due to previous poor scan experience. Females were more likely to decline MRI, but the sample is too small to draw conclusions, as reasons varied across both sexes (Fig. 1). All adolescents with a clinical diagnosis of NeuP were recruited irrespective of underlying cause, but several with complex or multiple types of pain were excluded from the MRI phase of the study. Refining inclusion/exclusion criteria to reduce heterogeneity in larger cohorts of adolescents with NeuP remains challenging. Current results may not generalize to studies with longer scanning protocols or task-based fMRI studies. Use of standardized postscan scales will facilitate comparison across studies.^33^

## 6. Conclusion

Research MRI is feasible and acceptable for most adolescents with moderate–severe NeuP.

## Disclosures

The authors have no conflicts of interest to declare.

This research was supported by funds from Great Ormond Street Hospital Children's Charity Research Awards W1071H, W1071I (S.M.W.), and a University College London—University of Toronto Joint Research Project and Exchange Activities Award (C.A.C., M.M., M.V., and S.M.W.).

Research at Great Ormond Street Hospital NHS Foundation Trust and UCL Great Ormond Street Institute of Child Health is supported by the NIHR Great Ormond Street Hospital Biomedical Research Centre. The views expressed are those of the author(s) and not necessarily those of the NHS, the NIHR or the Department of Health.

Sections of this manuscript were presented in poster form at the 7th International Congress on Neuropathic Pain (NeuPSIG 2019; May 9–11, 2019).

## Appendix A. Supplemental digital content

Supplemental digital content associated with this article can be found online at http://links.lww.com/PR9/A60.

## Supplementary Material

SUPPLEMENTARY MATERIAL
