# Supplementary material for: The feasibility and acceptability of research magnetic resonance imaging in adolescents with moderate–severe neuropathic pain
Source: Pain Rep. 2020 Jan 21;5(1):e807. doi: 10.1097/PR9.0000000000000807 (PMC7004507; doi:10.1097/PR9.0000000000000807)
Supplement: SUPPLEMENTARY MATERIAL [file painreports-5-e807-s001.pdf]

Supplementary Figure 1. Experience and acceptability questionnaire completed by patients following brain neuroimaging.

UCL GREAT ORMOND STREET INSTITUTE OF CHILD HEALTH

Great Ormond Street Hospital for Children NHS
NHS Foundation Trust

Neuropathic Pain   Patient Acceptability Questionnaire - BRAIN IMAGING   IRAS: 226141

STUDY NUMBER..........................

We would like to ask you some questions about your experience during the brain scan.

Please circle the number that matches your answer to each question.

Did you experience any discomfort during the brain scan?

0 1 2 3 4 5 6 7 8 9 10

Not at all — Very much so

Were you worried during the brain scan?

0 1 2 3 4 5 6 7 8 9 10

Not at all — Very much so

Do you think there are any risks for you in having a brain scan?

0 1 2 3 4 5 6 7 8 9 10

Not at all — Very much so

Were the instructions during the brain scan easy to understand?

0 1 2 3 4 5 6 7 8 9 10

Not at all — Very much so

Overall, do you think it is ok for a brain scan to be performed to help understand "nerve" pain in children?

0 1 2 3 4 5 6 7 8 9 10

Not at all — Very much so

Would you be happy to have a brain scan again in the future as part of your medical care?

0 1 2 3 4 5 6 7 8 9 10

Not at all — Very much so

Would you be happy to have a brain scan again in the future for medical research purposes?

0 1 2 3 4 5 6 7 8 9 10

Not at all — Very much so

Do you want to tell us anything else about the brain scan test?

____

____

____

V1, 12 April 2017
