# Supplementary material for: The feasibility and acceptability of research magnetic resonance imaging in adolescents with moderate–severe neuropathic pain
Source: Pain Rep. 2020 Jan 21;5(1):e807. doi: 10.1097/PR9.0000000000000807 (PMC7004507; doi:10.1097/PR9.0000000000000807)
Supplement: SUPPLEMENTARY MATERIAL [file painreports-5-e807-s002.pdf]

Supplementary Figure 2. Experience and acceptability questionnaire completed by parents following their child's brain neuroimaging scan.

UCL GREAT ORMOND STREET INSTITUTE OF CHILD HEALTH

Great Ormond Street Hospital for Children NHS Foundation Trust

Neuropathic Pain Parent Acceptability Questionnaire - BRAIN IMAGING IRAS: 226141

STUDY NUMBER……………………….

We would like to ask you some questions about your child's brain scanning test.

Please circle the number that matches your answer to each question.

Did your child experience any discomfort during the brain scan?

0 1 2 3 4 5 6 7 8 9 10

Not at all Very much so

To what extent do you think there might be risks in having a brain scan?

0 1 2 3 4 5 6 7 8 9 10

Not at all Very much so

Overall, did you find the brain scan was an acceptable test for your child?

0 1 2 3 4 5 6 7 8 9 10

Not at all Very much so

Would you be happy for your child to have a brain scan again in the future for clinical purposes?

0 1 2 3 4 5 6 7 8 9 10

Not at all Very much so

Would you be happy for your child to have a brain scan again in the future for medical research purposes?

0 1 2 3 4 5 6 7 8 9 10

Not at all Very much so

Do you want to tell us anything else about the brain scanning test?

____

____

____

V1, 12 April 2017
