# Supplementary material for: The feasibility and acceptability of research magnetic resonance imaging in adolescents with moderate–severe neuropathic pain
Source: Pain Rep. 2020 Jan 21;5(1):e807. doi: 10.1097/PR9.0000000000000807 (PMC7004507; doi:10.1097/PR9.0000000000000807)
Supplement: SUPPLEMENTARY MATERIAL [file painreports-5-e807-s003.docx]

**Supplementary Methods**

*Manuscript* *Title:*

**The feasibility and acceptability of research magnetic resonance imaging in adolescents with moderate-severe neuropathic pain**

*Manuscript Authors:*

Madeleine Verriotis, Massieh Moayedi, Clarissa Sorger, Judy Peters, Kiran Seunarine, Christopher A. Clark, and Suellen M. Walker

**1. Supplementary Methods**

*1.1 Recruitment for MRI and analysis of consent rate*

The Pain Service at Great Ormond Street Hospital NHS Trust has a specialist interest in neuropathic pain (average 30% of 250 new chronic pain referrals per year). Clinic patients aged between 10 and 18 years with clinical diagnosis (based on history, pain descriptors, and examination) of chronic NeuP, referred by experienced paediatric pain physicians for Quantitative Sensory Testing (QST), were eligible for inclusion in the neuropathic pain study (clinicaltrials.gov NCT03312881; West Midlands Research Ethics Committee Approval 17/WM/0306; 23-7-2017). Adolescents were excluded if they had significantly impaired comprehension (less than school level for 10 year old) or inadequate English language skills, as questionnaires are validated in English and sensory testing instructions could only be delivered by the investigators in English. Study information was posted to eligible families at least a few weeks prior to meeting the research team. Adolescents were approached for consent following routine QST appointments.

Within the 18-month recruitment period (10/2017–04/2019), 124 patients with chronic pain were referred for QST and 54 were eligible for the study (i.e. were 10-18 years old and had a clinical diagnosis of NeuP). Of these, 2 families did not wish to discuss the study due to time constraints and 2 declined all components of the study.

Families wishing to participate in the study gave consent to inclusion of their information from the clinical record in the study, including PROMs and QST results. The consent form included the option to additionally consent to a brain MRI scan for research purposes. Adolescents younger than 11 years were not approached for consent for the MRI part of the study. The MRI consent rate was calculated as a proportion of those families consenting to the overall study.

*1.2 MRI Exclusion criteria*

Following consent, to reduce heterogeneity within our pilot MRI study, we excluded patients whose symptoms would limit attribution of MRI changes (relative to control adolescents) to current NeuP. This comprised patients without clear predominant NeuP features on QST testing, those with multiple types of pain, and those whose symptoms had improved by the time of QST testing; we also excluded those not currently attending hospital appointments (Fig. 1). Current ICD-11 guidelines for chronic NeuP emphasise the role of neurophysiological testing, and that objective signs of a sensory disorder in the distribution of pain increase diagnostic certainty [2]. In accordance with NeuPSIG guidelines [1], QST findings were considered consistent with NeuP if they included a mixed pattern of sensory loss and gain across multiple modalities. QST assessments involved a standardised protocol with a range of stimulus modalities and intensities, including detection of thermal and mechanical stimuli, as described previously [3].

Exclusion criteria also included patients with MRI or comorbidity contraindications, including (i) claustrophobia; (ii) pregnancy; (iii) magnetic implants of any type; and (iv) significant medical problems such as uncontrolled asthma or seizure disorder, acute cardiac disease, psychiatric problems and other (non-neuropathic) neurological disease.

The remaining participants were eligible for the MRI part of the study, and families were contacted to arrange an MRI scan on a date within 3 months of their QST appointment. Despite agreeing to a date, two families did not attend their MRI appointment and were not contacted again, due to the 3-month time constraint.

*1.3 MRI acquisition*

Given our paediatric cohort, the MRI protocol was restricted to a maximum of 30 minutes, including preparation time in between acquisitions. Care was taken during initial preparation of participants to explain the MRI scan procedure and to minimise positional discomfort (with the use of under-arm and under-knee pillows and padding around the head, as required) and noise discomfort (using ear-plugs and headphones) during the scan. Participants watched a movie of their choice during structural acquisitions, which was turned off during the resting state acquisition. Age-appropriate instructions were given throughout scanning (e.g. during breaks in between acquisitions).

Anatomical T1-weighted scans were acquired with the following sequence: TE/TR=2.74/2300ms; time of inversion=909ms; 240 slices; flip angle=8°; in-plane matrix resolution=256 × 256 and field of view=256 x 256 mm, resulting in a voxel size=1x1x1mm; and GRAPPA acceleration factor=2. Total scan time = 5’35’’.

Diffusion-weighted scans were acquired with the following sequence: TE/TR= 60/3050ms; 66 slices; 0.2mm slice gap; flip angle=90°; in-plane matrix resolution=110 × 110 and field of view=220 x 220 mm, resulting in a voxel size=2x2x2mm; and multiband acceleration factor=2. In addition, a b0 image with a negated phase-encode direction was acquired for distortion correction. Total scan time = 7’50’’

rsfMRI scans were acquired with the following parameters: TE/TR= 26/1240ms; 40 slices; flip angle=75°; in-plane matrix resolution=80 × 80 and field of view=200 x 200 mm, resulting in a voxel size=2.5x2.5x2.5mm; 300 volumes; and multiband acceleration factor=2. Total scan time = 6’25’’

Field map images were acquired with the following parameters: TE/TR=10/1020ms; 40 slices; flip angle=90°; and in-plane matrix resolution=80 × 80 and field of view=200 x 200 mm, resulting in a voxel size=2.5x2.5x2.5mm. Total scan time = 2’47’’

*1.4 MRI preprocessing*

Briefly, preprocessing of fMRI scans involved motion- and distortion-correction with field maps, and slice-timing -correction. For this paediatric sample, outlier volumes were identified (for scrubbing) with liberal criteria (global signal z-value threshold=9, subject-motion threshold=2mm), using the Artifact Rejection Toolbox within CONN. Functional T2*-weighted scans were co-registered to the participant’s T1-weighted scan. Anatomical images were segmented for grey and white matter and cerebrospinal fluid, and were normalized to the MNI152 template. Data were smoothed with a Gaussian kernel of 8mm at full-width half-maximum (FWHM).

References[1] Baron R, Maier C, Attal N, Binder A, Bouhassira D, Cruccu G, Finnerup NB, Haanpaa M, Hansson P, Hullemann P, Jensen TS, Freynhagen R, Kennedy JD, Magerl W, Mainka T, Reimer M, Rice AS, Segerdahl M, Serra J, Sindrup S, Sommer C, Tolle T, Vollert J, Treede RD. Peripheral neuropathic pain: a mechanism-related organizing principle based on sensory profiles. Pain 2017;158(2):261-272.

[2] Scholz J, Finnerup NB, Attal N, Aziz Q, Baron R, Bennett MI, Benoliel R, Cohen M, Cruccu G, Davis KD, Evers S, First M, Giamberardino MA, Hansson P, Kaasa S, Korwisi B, Kosek E, Lavand'homme P, Nicholas M, Nurmikko T, Perrot S, Raja SN, Rice ASC, Rowbotham MC, Schug S, Simpson DM, Smith BH, Svensson P, Vlaeyen JWS, Wang SJ, Barke A, Rief W, Treede RD, Classification Committee of the Neuropathic Pain Special Interest G. The IASP classification of chronic pain for ICD-11: chronic neuropathic pain. Pain 2019;160(1):53-59.

[3] Walker SM, Melbourne A, O'Reilly H, Beckmann J, Eaton-Rosen Z, Ourselin S, Marlow N. Somatosensory function and pain in extremely preterm young adults from the UK EPICure cohort: sex-dependent differences and impact of neonatal surgery. Br J Anaesth 2018;121(3):623-635.
