# Supplementary material for: The feasibility and acceptability of research magnetic resonance imaging in adolescents with moderate–severe neuropathic pain
Source: Pain Rep. 2020 Jan 21;5(1):e807. doi: 10.1097/PR9.0000000000000807 (PMC7004507; doi:10.1097/PR9.0000000000000807)
Supplement: SUPPLEMENTARY MATERIAL [file painreports-5-e807-s004.doc]

**STROBE Statement—Checklist of items that should be included in reports of *cohort studies***

|  | Item No | Recommendation | Page reference and Details  Text taken directly from manuscript highlighted in *italics* |
| --- | --- | --- | --- |
| **Title and abstract** | 1 | (*a*) Indicate the study’s design with a commonly used term in the title or the abstract | Title: *The feasibility and acceptability of research magnetic resonance imaging in adolescents with moderate-severe neuropathic pain*  Abstract: ***prospective cohort study.*** |
|  |  | (*b*) Provide in the abstract an informative and balanced summary of what was done and what was found | Structured abstract within required word limit, with clear headings for background, objective, methods, results, and conclusions. |
| Introduction | | |  |
| Background/rationale | 2 | Explain the scientific background and rationale for the investigation being reported | p.1, Introduction section, paragraphs 1-2.  *Lack of evidence regarding feasibility and practical or ethical burden of MRI in such cohorts [28] may represent barriers to research study planning, ethical approval, and/or recruitment [33].* |
| Objectives | 3 | State specific objectives, including any prespecified hypotheses | *Within a larger clinical cohort of adolescents with moderate-severe NeuP, a pilot study assessed MRI consent rate, post-scan acceptability, and data quality.* |
| Methods | | |  |
| Study design | 4 | Present key elements of study design early in the paper | p.1, Methods section. *Adolescents aged 10-18 years with clinically diagnosed NeuP were recruited from the Great Ormond Street Hospital Chronic Pain Management Service. … Age-matched healthy participant data with the same MRI protocol and scanner were available for comparison.* |
| Setting | 5 | Describe the setting, locations, and relevant dates, including periods of recruitment, exposure, follow-up, and data collection | p.1, Methods section. *The MRI pilot forms part of an ongoing cohort study evaluating PROMs and QST. …* *families were given the option to additionally consent to an MRI scan, which required one additional hospital visit within 3 months of QST testing and recruitment*  p.2, Methods section. *Multimodal neuroimaging was performed using a 3T Siemens Prisma MRI scanner with a 64-channel coil at Great Ormond Street Hospital.*  Supplementary Material, p.2 *Within the 18-month recruitment period (10/2017–04/2019), 124 patients with chronic pain were referred for QST and 54 were eligible for the study* |
| Participants | 6 | (*a*) Give the eligibility criteria, and the sources and methods of selection of participants. Describe methods of follow-up | Fig. 1 Recruitment flow chart  Supplementary Material, p.2, para 1. *Clinic patients aged between 10 and 18 years with clinical diagnosis (based on history, pain descriptors, and examination) of chronic NeuP, referred by experienced paediatric pain physicians for Quantitative Sensory Testing (QST), were eligible for inclusion in the neuropathic pain study (clinicaltrials.gov NCT03312881; West Midlands Research Ethics Committee Approval 17/WM/0306; 23-7-2017). Adolescents were excluded if they had significantly impaired comprehension (less than school level for 10 year old) or inadequate English language skills, as questionnaires are validated in English and sensory testing instructions could only be delivered by the investigators in English. Study information was posted to eligible families at least a few weeks prior to meeting the research team. Adolescents were approached for consent following routine QST appointments.*  Supplementary Material, p.2, para 4. *Following consent, to reduce heterogeneity within our pilot MRI study, we excluded … patients without clear predominant NeuP features on QST testing, those with multiple types of pain, and those whose symptoms had improved by the time of QST testing; we also excluded those not currently attending hospital appointments* |
|  |  | (*b*) For matched studies, give matching criteria and number of exposed and unexposed | p.1, para 1. *Age-matched healthy participant data with the same MRI protocol and scanner were available for comparison.*  Table 2 summarises participant demographic data and demonstrates appropriate age match of patients with neuropathic pain and control participants. |
| Variables | 7 | Clearly define all outcomes, exposures, predictors, potential confounders, and effect modifiers. Give diagnostic criteria, if applicable | p. 1-2 Measures  Table 1: additional diagnosis details in patients with neuropathic pain.  Supplementary Material, p.2, para 4. *Current ICD-11 guidelines for chronic NeuP emphasise the role of neurophysiological testing, and that objective signs of a sensory disorder in the distribution of pain increase diagnostic certainty [2]. In accordance with NeuPSIG guidelines [1], QST findings were considered consistent with NeuP if they included a mixed pattern of sensory loss and gain across multiple modalities. QST assessments involved a standardised protocol with a range of stimulus modalities and intensities, including detection of thermal and mechanical stimuli, as described previously [3].* |
| Data sources/ measurement | 8* | For each variable of interest, give sources of data and details of methods of assessment (measurement). Describe comparability of assessment methods if there is more than one group | p. 1-2 Measures.  Additional details relating to MRI acquisition parameters and preprocessing methods are detailed and referenced in Supplementary Material. |
| Bias | 9 | Describe any efforts to address potential sources of bias | p.1 *Age-matched healthy participant data with the same MRI protocol and scanner were available for comparison* |
| Study size | 10 | Explain how the study size was arrived at | The primary aim of the pilot MRI study within the overall descriptive cohort study was to assess feasibility of research MRI in adolescents with moderate-severe neuropathic pain, and estimate recruitment rate. Therefore, we aimed to recruit the maximum available subjects, and no a priori power analysis was performed. |
| Quantitative variables | 11 | Explain how quantitative variables were handled in the analyses. If applicable, describe which groupings were chosen and why | p. 2 MRI acquisition and analysis. *FD values were compared between adolescents with NeuP and controls.*  p. 2 Data analyses  The following measures are descriptive within the cohort of adolescents with neuropathic pain:  Supplementary Material, p.2, para 3. *The MRI consent rate was calculated as a proportion of those families consenting to the overall study*.  Mean (SD) demographic characteristics, pain ratings, and questionnaire scores are reported in Table 1. Range of acceptability and discomfort ratings are presented in the text and Fig. 2. |
| Statistical methods | 12 | (*a*) Describe all statistical methods, including those used to control for confounding | p. 2 MRI acquisition and analysis.  p. 2 Data analyses  Figure and Table Legends include additional details.  Supplementary Material, p.3, para 1 |
|  |  | (*b*) Describe any methods used to examine subgroups and interactions | Figure 3 presents correlation between head motion and age per group; details in Legend. |
|  |  | (*c*) Explain how missing data were addressed | The only variables with missing data are questionnaire measures. Table 1 presents mean questionnaire scores, and the Legend indicates number of participants completing questionnaire measures |
|  |  | (*d*) If applicable, explain how loss to follow-up was addressed | n/a (not a longitudinal design) |
|  |  | (*e*) Describe any sensitivity analyses | n/a |
| Results | | |  |
| Participants | 13* | (a) Report numbers of individuals at each stage of study—eg numbers potentially eligible, examined for eligibility, confirmed eligible, included in the study, completing follow-up, and analysed | Figure 1  Supplementary Material, p.2-3 provides further details on eligibility and exclusion criteria, including number potentially eligible |
|  |  | (b) Give reasons for non-participation at each stage | Figure 1 presents all reasons for non-participation at each stage  Supplementary Material, p.2-3 provides further details on eligibility and exclusion criteria, |
|  |  | (c) Consider use of a flow diagram | Figure 1 is presented as a flow diagram of participant numbers at each stage of the study. |
| Descriptive data | 14* | (a) Give characteristics of study participants (eg demographic, clinical, social) and information on exposures and potential confounders | Tables 1 & 2 |
|  |  | (b) Indicate number of participants with missing data for each variable of interest | The only variables with missing data are questionnaire measures. Table 1 presents mean questionnaire scores, and the Legend indicates number of participants completing these questionnaire measures |
|  |  | (c) Summarise follow-up time (eg, average and total amount) | The study involved a single study visit:  Supplementary Material, p.2, para 1. *Adolescents were approached for consent following routine QST appointments*  p.1, para 3. *families were given the option to additionally consent to an MRI scan, which required one additional hospital visit within 3 months of QST testing and recruitment*  p.1, para 4. *At recruitment, adolescents completed Visual Analogue Scales (VAS; 0-10cm) for pain intensity (now, average and worst pain in the last week) and activity interference due to pain [40]. Twelve adolescents also reported pain intensity immediately prior to MRI.*  p.2, para 2. *Following the scan, adolescents and parent(s) rated discomfort, perceived risk, and acceptability of current and future MRI scans.* |
| Outcome data | 15* | Report numbers of outcome events or summary measures over time | The study involved a single study visit for a research MRI scan, pain ratings, and post-MRI questionnaires, which form the main variables of interest in the study (see also 14c).  Additional descriptive and demographic data were collected from the clinical record (pain ratings and questionnaires). |
| Main results | 16 | (*a*) Give unadjusted estimates and, if applicable, confounder-adjusted estimates and their precision (eg, 95% confidence interval). Make clear which confounders were adjusted for and why they were included | Fig.3 data are expressed with 95% confidence interval. |
|  |  | (*b*) Report category boundaries when continuous variables were categorized | n/a |
|  |  | (*c*) If relevant, consider translating estimates of relative risk into absolute risk for a meaningful time period | n/a |
| Other analyses | 17 | Report other analyses done—eg analyses of subgroups and interactions, and sensitivity analyses | n/a |
| Discussion | | |  |
| Key results | 18 | Summarise key results with reference to study objectives | p.4 para 1of Discussion  p.5 para 2 of Discussion |
| Limitations | 19 | Discuss limitations of the study, taking into account sources of potential bias or imprecision. Discuss both direction and magnitude of any potential bias | p.5-6, Limitations section: *The number of adolescents scanned for this pilot study is small (n=21), and the MRI acceptability questionnaire was introduced after the first 3 participants. Acceptability ratings do not account for potential lower scores in 3 participants who declined due to previous poor scan experience. Females were more likely to decline MRI, but the sample is too small to draw conclusions, as reasons varied across both sexes (Fig. 1). All adolescents with a clinical diagnosis of NeuP were recruited irrespective of underlying cause, but several with complex or multiple types of pain were excluded from the MRI phase of the study. Refining inclusion/exclusion criteria to reduce heterogeneity in larger cohorts of adolescents with NeuP remains challenging. Current results may not generalize to studies with longer scanning protocols or task-based fMRI studies. Use of standardized post-scan scales will facilitate comparison across studies [33].* |
| Interpretation | 20 | Give a cautious overall interpretation of results considering objectives, limitations, multiplicity of analyses, results from similar studies, and other relevant evidence | p. 4-6: Discussion |
| Generalisability | 21 | Discuss the generalisability (external validity) of the study results | p. 5-6. Limitations section: *Current results may not generalize to studies with longer scanning protocols or task-based fMRI studies. Use of standardized post-scan scales will facilitate comparison across studies [33].* |
| Other information | | |  |
| Funding | 22 | Give the source of funding and the role of the funders for the present study and, if applicable, for the original study on which the present article is based | p. 7 Acknowledgements.  *This research was supported by funds from Great Ormond Street Hospital Children’s Charity Research Awards W1071H, W1071I (S.M.W.) and a University College London – University of Toronto Joint Research Project and Exchange Activities Award (C.A.C., M.M., M.V., S.M.W.).* |

*Give information separately for exposed and unexposed groups.

**Note:** An Explanation and Elaboration article discusses each checklist item and gives methodological background and published examples of transparent reporting. The STROBE checklist is best used in conjunction with this article (freely available on the Web sites of PLoS Medicine at http://www.plosmedicine.org/, Annals of Internal Medicine at http://www.annals.org/, and Epidemiology at http://www.epidem.com/). Information on the STROBE Initiative is available at http://www.strobe-statement.org.
